# UV-C Irradiation Delays the Quality Deterioration of Postharvest Shiitake Mushrooms (*Lentinula edodes*)

**DOI:** 10.3390/foods15111908

**Published:** 2026-05-28

**Authors:** Yunyun Han, Run Deng, Shaojun Zhang, Li Zhang, Yu Wang

**Affiliations:** 1 College of Horticulture, Shanxi Agricultural University, Taigu, Jinzhong 030801, China; hanyunyun_x@163.com; 2 College of Resources and Environmental Science, Shanxi Agricultural University, Taigu, Jinzhong 030801, China; 3 College of Food Science and Engineering, Shanxi Agricultural University, Taigu, Jinzhong 030801, China; 18835105409@163.com (R.D.); z18835028855@163.com (S.Z.); zhang_stream@163.com (L.Z.); 4 Xinzhou Comprehensive Inspection and Testing Center, Xinzhou 034000, China

**Keywords:** *Lentinula edodes*, UV-C irradiation, postharvest quality, preservation, cell wall degradation

## Abstract

The fruiting bodies of shiitake mushrooms (*Lentinula edodes*) are highly susceptible to postharvest quality deterioration due to their loose tissue, high moisture, and active metabolism. Low-dose ultraviolet-C (UV-C) irradiation, a green, safe, low-cost, and efficient physical treatment especially suitable for high-moisture and metabolically active produce, offers a promising solution to this problem for the postharvest preservation of shiitake mushrooms. In this study, the effects of different doses of UV-C irradiation (2.5, 5.0, and 7.5 kJ·m^−2^) on the postharvest quality of shiitake mushrooms were systematically compared by measuring weight loss, respiration intensity, cap opening percentage, firmness, color (L*, a*, b*), electrolyte leakage, MDA content, antioxidant enzyme activities (SOD, CAT, POD, PAL), chitin content, chitinase activity, and the expression of *LeCHI* and *LeCDA* genes. The results showed that UV-C irradiation at 5 kJ m^−2^ significantly delayed the increase in weight loss (by approximately 32% at day 15 compared to control) and reduced respiration rate (by 25–35% during days 3–9) and cap opening rate (27.3% vs. 48.0% in control at day 15) while maintaining higher firmness and better color retention over the control group. Furthermore, this treatment effectively inhibited electrolyte leakage and malondialdehyde accumulation, enhanced the activities of SOD, POD, CAT, and PAL, maintained chitin content, and downregulated the expression of chitinase and cell wall degradation-related genes. Thus, 5 kJ m^−2^ UV-C irradiation is effective in maintaining postharvest quality and extending the storage time of shiitake mushrooms.

## 1. Introduction

As a key basidiomycete, *Lentinula edodes* (shiitake mushroom) accounts for 30.08% of China’s total edible fungus production and dominates the edible fungus market [1]. In recent years, as the global consumption pattern of edible fungi has shifted rapidly from dried to fresh products, more than 85% of shiitake mushrooms are now marketed as fresh produce. However, fresh shiitake mushrooms exhibit extremely active postharvest metabolism, making them highly susceptible to quality deterioration, primarily tissue softening and surface browning, which causes substantial economic losses [2].

Tissue softening in edible fungi arises mainly from structural changes and degradation of cell wall components, representing a sophisticated physiological process. Unlike plants, the cell wall of edible fungi mainly contains chitin and glucans, with chitin forming a microfibril skeleton network and glucans (predominantly formed by β-1,3- and β-1,6-glucans) filling the spaces as a gel matrix to provide mechanical strength. During postharvest storage, the activities of cell wall hydrolases (e.g., chitinase, glucanase) increase, leading to degradation of chitin and glucans and subsequent tissue softening [3]. In addition, postharvest browning of horticultural products is closely associated with oxidative stress, generally driven by membrane lipid peroxidation triggered by an overabundance of reactive oxygen species (ROS) within tissues. The consequent increase in malondialdehyde (MDA) content disrupts membrane integrity and eventually induces browning. To counteract this process, a system has been developed in organisms, which scavenges excessive ROS through antioxidant enzymes, thereby keeping cellular ROS levels in check [4,5,6].

Notably, the fruiting body of edible fungi is formed by intertwined hyphae, featuring a loose internal structure and lacking the protection of peel tissue and cuticle. When treated by immersion in preservative solutions, the tissue readily absorbs water and swells, which greatly limits the application of immersion-based postharvest treatments for edible fungi. Ultraviolet-C (UV-C) irradiation is a mild, non-immersion physical treatment that can activate the defense system of fruits and vegetables, induce the accumulation of phenolic compounds and increase the activities of disease-resistance-related enzymes, while also partially inhibiting cell-wall-degrading enzyme activities, thereby helping to maintain postharvest texture [7,8,9,10,11,12]. In edible fungi, numerous studies have confirmed that UV-C irradiation improves the color and firmness of edible fungi such as *Hypsizygus marmoreus*, *Pleurotus eryngii*, *Agaricus bisporus*, *Pleurotus ostreatus*, and *Naematelia aurantialba*, reduces MDA accumulation during storage, and delays quality deterioration [13,14,15,16,17]. In addition, UV-C irradiation induces the activities of phenylalanine ammonia-lyase (PAL) and catalase (CAT) in the organism, enhances the antioxidant defense system, and significantly inhibits postharvest tissue softening and browning in *P. ostreatus* [15].

Despite these previous studies, important knowledge gaps remain. Most reports have focused only on general antioxidant responses and basic quality parameters (firmness, color) of edible fungi, without investigating the cell wall polysaccharide metabolism or its molecular regulation [13,14,15,16]. In particular, no study has examined the effect of UV-C on chitin content, chitinase activity, and the expression of chitin-related genes (*LeCHI*, *LeCDA*) in edible fungi. Furthermore, dose optimization has rarely been performed, with most studies using a single fixed dose without comparing multiple levels or verifying the hormetic zone [6,7,8,9,13]. To address these gaps, the present study systematically compared three UV-C doses (2.5, 5.0, and 7.5 kJ·m^−2^) and evaluated, for the first time, the integrated effects on membrane integrity, antioxidant enzyme system, and chitin metabolism at both the biochemical and transcriptional levels. We specifically tested the hypothesis that a properly optimized UV-C dose maintains postharvest quality by suppressing cell wall degradation via regulation of chitinase gene expression, in addition to enhancing antioxidant defense.

In addition, excessive or high-dose UV-C irradiation can induce detrimental effects on postharvest quality, primarily through oxidative stress and tissue damage. Prolonged exposure disrupts cellular homeostasis, leading to increased membrane permeability, lipid peroxidation, and enzymatic browning in lettuce [18]. These adverse responses manifest as surface discoloration, textural degradation, accelerated water loss, and reduced nutritional value [19,20]. For example, high-intensity UV-C has been reported to stimulate ethylene production and respiratory activity in some horticultural products, accelerating senescence instead of delaying it [21]. In mushrooms, the limited penetration depth of UV-C (mainly affecting the outermost cell layers) means that overdosing can scorch the cap surface without providing additional preservation benefits. Therefore, dose optimization is critical to achieving a hormetic effect—where a low dose triggers protective stress responses while a high dose causes irreversible injury. In this study, we systematically compared three UV-C doses (2.5, 5.0, and 7.5 kJ·m^−2^) on shiitake mushrooms, paying particular attention to the negative consequences of excessive irradiation. We focused on evaluating membrane integrity (electrolyte leakage, MDA content), antioxidant enzyme activities, as well as the activity of the tissue-softening enzyme (chitinase) and its corresponding gene expression level. Through this multi-level analysis, we aimed to expand the understanding of how UV-C maintains postharvest quality by regulating oxidative stress and cell wall metabolism, and to provide a theoretical basis for developing dose-optimized, green preservation strategies for edible fungi.

## 2. Materials and Methods

### 2.1. Mushroom and Postharvest Treatment

Shiitake mushrooms were harvested from a commercial mushroom cultivation facility named Shanxi Huitao Technology Co., Ltd. (Lin County, Luliang, China) and immediately transported to the laboratory after harvesting. Mushroom fruiting bodies of uniform size, identical developmental stage, with unopened caps, and free from mechanical damage, pest infestation and disease infection were selected. A UV-C lamp was positioned 35 cm above the samples, providing an irradiation intensity of 248 μW cm^−2^ at that distance. Mushrooms were arranged in a single layer with the cap facing upward. The uniformity of UV-C irradiance across the sample area was verified by measuring nine evenly spaced positions using a UV radiometer (UVC254, Sanpometer, Shenzhen, China). Only mushrooms of uniform size with cap diameters of 5–6 cm and convex unopened caps were selected to minimize morphological variation. Based on different irradiation durations, four treatment groups were established: a control group (no irradiation), and three UV-C treatment groups receiving 2.5, 5.0, and 7.5 kJ m^−2^ (irradiation durations of 17, 34, and 51 min), respectively. After irradiation, the mushrooms were stored at (4 ± 1) °C and 85% RH. Every two days, samples were collected before each treatment for measurement of various physiological and biochemical indices. The cap samples were sliced into small pieces and instantly frozen in liquid nitrogen and stored at −86 °C for further analyses.

### 2.2. Determination of Color

Surface L*, a* and b* values of shiitake mushrooms were measured using a WSC-C colorimeter (Shanghai Precision Scientific Instrument Co., Ltd., Shanghai, China). Nine mushrooms were randomly selected from each experimental group as fixed samples for measurement, and two fixed positions on the surface of each mushroom were marked for continuous monitoring.

### 2.3. Determination of Cap Opening, Weight Loss, and Respiration Rate

Fifty-one mushrooms were randomly selected from each experimental group as fixed samples and further randomly divided into three subgroups, with 17 mushrooms per subgroup. The cap opening was calculated as:

(1) Cap opening (%) = (Number of mushrooms with open caps/17) × 100%

The measurement of weight loss referred to the method of Deng et al. [22]. Twelve mushrooms were randomly selected from each experimental group as fixed samples and randomly divided into three subgroups. The total weight of each subgroup was measured every two days, and the weight loss was calculated as:

(2) Weight loss (%) = (w_0_ − w_1_)/w_0_ × 100%

where w_0_ is the initial total weight (g) of the subgroup, and w_1_ is the total weight (g) of the subgroup on the day of measurement (day 0, 3, 6, 9, 12, 15).

The determination of respiratory intensity followed the method of Deng et al. [22]. Thirty mushrooms from each treatment group were randomly divided into three subgroups, with 10 mushrooms per subgroup. Each subgroup was placed in a 4500 mL sealed container. After being kept sealed and static for 2 h, the CO_2_ concentration inside the container was measured using an F-940 portable gas analyzer (Felix Instruments, Inc., Camas, WA, USA). Respiratory intensity was calculated using the formula:

(3) Respiratory intensity (mg CO_2_ kg^−1^ h^−1^) = [(c_1_ − c_0_) × (4500 − m) × 44 × 1000]/(22.4 × m × T)

where c_1_ is the initial CO_2_ concentration in air (%), c_0_ is the CO_2_ concentration inside the container after being sealed for time T (%), m is the total mass of the mushroom samples (g), and T is the sealing time (h). In addition, the 4500, 44, 1000 and 22.4 in this formula mean the volume of the sealed container, the relative molecular mass of CO_2_ (g mol^−1^), the conversion factor used to unify units and the universal gas constant (L mol^−1^), respectively.

### 2.4. Determination of Texture

Texture profile analysis (TPA) was carried out as described by Živanović et al. with some modifications [23]. A TA.XT Plus texture analyzer (Stable Micro Systems, Surrey, UK) was used. Fifteen mushroom samples were randomly selected and equally divided into three groups (5 mushrooms per group), and the stipes were removed. The samples were placed with the caps facing upward at the center of the test platform, and measurements were carried out using a cylindrical probe (P/5, φ = 5 mm) with a testing rate of 2 mm s^−1^. Hardness was defined as the maximum peak force during compression, and springiness as the distance the sample recovered after the first compression.

### 2.5. Determination of Electrical Conductivity and MDA Content

Electrical conductivity assays were conducted as described by Yu et al. [24]. Approximately 2 g of shiitake mushroom cap tissue was placed in an Erlenmeyer flask, and 30 mL of distilled water was added. The initial electrical conductivity (P_0_) was measured using a conductivity meter. The flask was then kept in a thermostatic shaker at 25 °C for 1 h, and the electrical conductivity (P_1_) was measured again. Finally, the sample was boiled for 30 min, cooled to 25 °C, and the final electrical conductivity (P_2_) was measured. Relative electrical conductivity was calculated using the following formula:

(4) Electrical conductivity (%) = (P_1_ − P_0_)/(P_2_ − P_0_) × 100

MDA was detected using the method reported by Hodges et al., with some modifications [25]. Exactly 1.0 g of shiitake mushroom cap tissue was accurately weighed, homogenized with 5 mL of 10% trichloroacetic acid solution, and then centrifuged at 4500–5000 rpm for 15 min. Subsequently, the supernatant (4 mL) was mixed with an equal volume of 0.6% thiobarbituric acid solution and heated at 100 °C for 15 min, then cooled to 25 °C and subjected to a second centrifugation, after which the supernatant was collected and its absorbances at 450 nm, 532 nm, and 600 nm were measured using a microplate reader. The MDA content was calculated and expressed in nmol per gram (nmol g^−1^).

(5) MDA content = [6.45 × (OD 532 − OD 600) − 0.56 × OD 450] × (5/4) × (1/1.0)

where the factors 5 and 4 denote the total extraction volume (mL) and the aliquot volume of the supernatant used for the reaction (mL), respectively, and 1.0 is the sample fresh weight (g).

### 2.6. Determination of SOD, CAT, POD and PAL Activities

Superoxide dismutase (SOD) activity was determined using a commercial kit (Solarbio, Beijing, China; Cat. No. BC5255). The assay was based on the inhibition of formazan reduction by superoxide anions generated by xanthine/xanthine oxidase, following the method of Peskin and Winterbourn [26]. Cap tissue (0.1 g) was homogenized with 0.9 mL of normal saline and centrifuged. The supernatant was collected and diluted to an appropriate concentration with normal saline, and the absorbance at 569 nm was subsequently recorded with a microplate reader (Thermo Fisher, Waltham, MA, USA) as per the kit instructions, with SOD activity expressed in U g^−1^.

CAT activity was measured using a commercial kit (Tiangen, Beijing, China; Cat. No. BC0205). The assay, according to the decrease in absorbance at 240 nm due to H_2_O_2_ decomposition, was monitored according to Johansson and Borg [27]. The sample pretreatment method was the same as that for SOD, and then the absorbance at 240 nm was measured using a microplate reader (Thermo Fisher, Waltham, MA, USA). CAT was expressed as U g^−1^.

Peroxidase (POD) activity was determined using the guaiacol method [28]. Mushroom tissue (1.0 g) was weighed, an extraction solution was added at a ratio of 1:10 (g mL^−1^), and the mixture was homogenized and centrifuged to obtain the supernatant. For the assay, an appropriate volume of the supernatant was mixed with 0.05 mol L^−1^ guaiacol solution and pre-incubated in a water bath at 30 °C for 5 min. Then, 0.2 mL of 0.2% H_2_O_2_ solution was added to initiate the reaction, and the change in absorbance at 470 nm was immediately monitored continuously for 3 min. POD was expressed as U g^−1^.

PAL activity was measured using a commercial kit (Solarbio, Beijing, China; Cat. No. BC0215). The assay was based on the formation of trans-cinnamic acid from L-phenylalanine, as described by Cheng [29]. A 0.2 g sample was extracted with 1 mL buffer in an ice bath and centrifuged at 10,000 *g* for 10 min. Subsequently, the absorbance of the supernatant at 290 nm was recorded using a microplate reader (Thermo Fisher, Waltham, MA, USA). PAL was expressed as U g^−1^.

### 2.7. Determination of Chitin Content and Chitinase Activity

Chitin content was determined according to the method of Fu et al. [30]. A 2 g sample was mixed with 2 mol L^−1^ hydrochloric acid solution (5 mL), followed by the addition of concentrated sulfuric acid to a final concentration of 75% (v v^−1^). The mixture was heated at 100 °C for 30 min and then diluted to volume to obtain the hydrolysate. Then, the hydrolysate (1 mL) was mixed sequentially with 2% resorcinol solution (1 mL) and 75% sulfuric acid solution (7.5 mL). After thorough shaking and mixing, the mixture was heated at 100 °C for 30 min. Following cooling to 25 °C, the reaction solution was diluted to 10 mL, and the absorbance of the supernatant at 500 nm was recorded using a microplate reader (Thermo Fisher, Waltham, MA, USA). Using a standard curve, the results were expressed as the content of D-glucosamine hydrochloride per gram of dry sample (mg g^−1^).

Chitinase activity was assayed with a commercial kit (Solarbio, Beijing, China; Cat. No. BC0825) by determining N-acetylglucosamine release from colloidal chitin using the 3,5-dinitrosalicylic acid (DNS) method [31]. A 0.2 g of sample was extracted with buffer (1 mL) in an ice bath and centrifuged at 10,000 *g* for 20 min. Subsequently, the absorbance of the supernatant at 585 nm was recorded using a microplate reader (Thermo Fisher, Waltham, MA, USA). Chitinase activity was expressed as U g^−1^.

### 2.8. Relative Expression Level Analysis of Target Genes

Total RNA was extracted according to the instructions of the TransZol Up Plus RNA Kit (TransGen Biotech, Beijing, China), based on the principle of guanidinium-thiocyanate-phenol-chloroform extraction [32]. Using the extracted total RNA as a template, cDNA synthesis was performed on a PCR thermal cycler following the protocol of the PrimeScript™ RT Reagent Kit with gDNA Eraser (TransGen Biotech, Beijing, China).

The primer sequences used in qRT-PCR were designed via TBtools software (version 2.351) and synthesized by Xi’an Tsingke Biotechnology Co., Ltd. (Xi’an, China). The reaction system (20 μL) consisted of 0.5 μL each of forward and reverse primers (10 μM), approximately 70 ng of cDNA template, 10 μL of SYBR Green PCR Supermix (TransGen Biotech, Beijing, China), and ddH_2_O to make up the final volume. The qRT-PCR was performed on a BIO-RAD CFX9600 real-time PCR system under the following program: a 2 min denaturation step at 95 °C, then 35 amplification cycles (95 °C for 5 s, 55 °C for 10 s, 72 °C for 40 s), and a final holding stage at 12 °C. After the reaction, melting curve analysis was performed. α-TUB was used as the reference gene for relative quantification.

### 2.9. Statistical Analysis

Experimental data were collated with Microsoft Excel 2021 and statistically analyzed using SPSS Statistics 27. All experiments were performed in triplicate, and the results were expressed as mean ± SD. Data were subjected to one-way analysis of variance (ANOVA), and Duncan’s multiple range test was used to determine significant differences among treatment means. Graphical representations were prepared with GraphPad Prism 9.5.

## 3. Results

### 3.1. Changes in Physical Appearance and Surface Color

**Figure 1:**
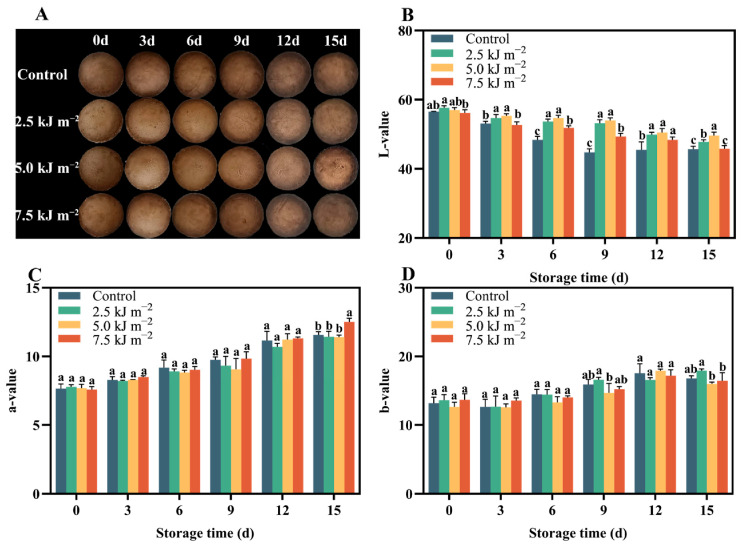
Postharvest changes in shiitake mushroom treated with UV-C. (**A**) Appearance, (**B**) L* value, (**C**) a* value, (**D**) b* value. Data are presented as mean ± standard error (*n* = 3). Different lowercase letters above bars indicate significant differences (*p* < 0.05).

The changes in physical appearance and surface luminosity (L*) of shiitake mushrooms are presented in Figure 1. After harvest, the surface color of shiitake mushrooms became progressively darker over time in the UV-C irradiation groups as well as in the control group (Figure 1A). Notably, while all UV-C doses tested resulted in significantly brighter surface coloration than the control, shiitake mushrooms treated with 5.0 kJ·m^−2^ UV-C exhibited significantly less cap browning from the mid-storage stage to the end of the storage period, compared not only to the control but also to all other UV-C dose groups. Furthermore, the surface L* values of shiitake mushrooms during postharvest storage were also examined (Figure 1B). Although a gradual decline in L* values over time was observed for all treatments (including the control and all UV-C treatments), the 2.5 and 5.0 kJ·m^−2^ UV-C treatments consistently showed significantly higher L* values than the other groups at each storage stage, with the 5.0 kJ·m^−2^ treatment giving the highest L* value (49.6) at the conclusion of storage (day 15). The changes in a* (redness) and b* (yellowness) values are presented in Figure 1C,D, respectively. Overall, a* values gradually increased from approximately 7.6–7.8 at day 0 to 11.2–12.8 at day 15 across all treatment groups, indicating progressive surface reddening during storage. Similarly, b* values rose from about 12.5–14.5 at day 0 to 15.2–18.5 at day 15, reflecting increasing yellowness. However, no significant differences in a* or b* values were observed between the control and any UV-C treatment group at any time point (*p *> 0.05). These results suggest that while UV-C irradiation at 5 kJ·m^−2^ effectively retarded surface browning as measured by L* (lightness), it did not significantly alter the chromaticity coordinates a* and b* under our experimental conditions. These findings were in good agreement with the visual appearance changes presented in Figure 1A, confirming that UV-C irradiation effectively retards the surface browning of shiitake mushrooms.

### 3.2. Changes in Postharvest Physiological and Quality Attributes

Postharvest quality of edible fungi is closely associated with their physiological metabolic activities, among which respiration plays a dominant role by leading to water loss, nutrient consumption, and triggering tissue softening and cap opening—the most intuitive quality deteriorations indicative of mushroom senescence. Therefore, we analyzed the effects of different UV-C treatments on respiration rate, weight loss, and cap opening of shiitake mushrooms to evaluate their preservation efficacy.

**Figure 2:**
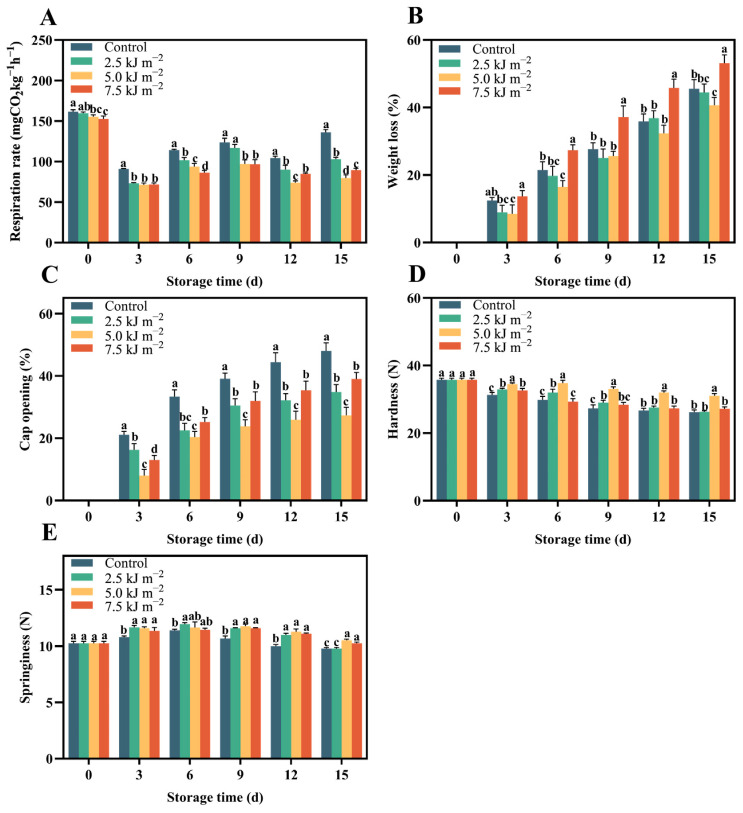
Effects of UV-C treatments on respiration rate (**A**), weight loss (**B**), cap opening (**C**), hardness (**D**) and springiness (**E**) of shiitake mushrooms stored at 0 °C for 15 days. Data are presented as mean ± standard error (*n* = 3). Different lowercase letters above bars indicate significant differences (*p* < 0.05).

The changes in respiration rate of shiitake mushrooms are shown in Figure 2A. Overall, upon transfer to the low-temperature environment (4 °C), the respiration rate of all treatment groups decreased significantly compared to day 0 and did not return to the initial level at any time point during storage. Interestingly, from day 3 to day 15, all UV-C treatment groups showed significantly lower respiration rates than the control group. Among them, the 7.5 kJ·m^−2^ UV-C treatment exhibited the lowest respiration rate from day 3 to day 6, while the 5.0 kJ·m^−2^ UV-C treatment maintained near the lowest levels from day 3 to day 9 and became significantly lower than all other treatments from day 12 to day 15.

We subsequently examined the changes in weight loss of shiitake mushrooms (Figure 2B). During postharvest storage, a steadily increasing trend in weight loss was observed across all treatment groups. Among them, the 7.5 kJ·m^−2^ UV-C treatment resulted in significantly higher weight loss than all other UV-C treatment groups and the control throughout the entire storage period. Notably, although this treatment exhibited the lowest respiration rate during the early storage period (days 3–6), its weight loss was the highest. This may be attributed to physical damage caused by the high UV-C dose, leading to disruption of cellular integrity, accelerated water loss, and simultaneously suppressed respiratory metabolism. In contrast, the 5.0 kJ·m^−2^ UV-C treatment showed the lowest weight loss at each storage stage, with statistically significant differences observed at days 3, 6, and 15 compared to the other groups.

The cap opening of all treatments showed a steadily increasing trend (Figure 2C) similar to that observed for weight loss. Specifically, at each storage stage, all UV-C treatments exhibited a significantly lower cap opening percentage than the control group. Throughout the entire storage period, the 5.0 kJ·m^−2^ UV-C treatment consistently resulted in the lowest cap opening percentage, reaching 20.4% at day 6, 23.8% at day 9, 25.9% at day 12, and 27.3% at day 15. In contrast, the control group showed the highest cap opening, which increased to 33.4%, 39.1%, 44.4%, and 48.0% at days 6, 9, 12, and 15, respectively. The 2.5 and 7.5 kJ·m^−2^ treatments exhibited intermediate levels, with final cap opening percentages of 34.8% and 39.0% at day 15. These findings indicate that UV-C irradiation at 5.0 kJ·m^−2^ is most effective in delaying cap opening, a key indicator of postharvest senescence in shiitake mushrooms.

The changes in hardness of shiitake mushrooms during postharvest storage are presented in Figure 2D. Overall, the hardness of all treatment groups decreased progressively with extended storage time. After three days of storage, the control group showed a marked decrease to 31.30 N, whereas the 2.5, 5.0, and 7.5 kJ·m^−2^ UV-C treatments maintained higher values of 32.93 N, 34.50 N, and 32.60 N, respectively. Notably, the 5.0 kJ·m^−2^ UV-C treatment consistently exhibited the highest hardness throughout the entire storage period, with values of 34.80 N at day 6, 33.10 N at day 9, 32.00 N at day 12, and 31.00 N at day 15. In contrast, the control group showed the most rapid softening, declining to 26.20 N by day 15. The 2.5 and 7.5 kJ·m^−2^ treatments showed intermediate hardness levels, with final values of 26.30 N and 27.20 N, respectively, at day 15. These results indicate that UV-C irradiation at 5.0 kJ·m^−2^ is most effective in retarding postharvest softening of shiitake mushrooms. Regarding springiness, in contrast to hardness, it initially increased from day 0 to days 3–6, with the 2.5 and 5.0 kJ·m^−2^ treatments peaking at 11.66 N and 11.65 N (day 6), then declined steadily to 9.78–10.52 N by day 15 (Figure 2E). At the end of storage, the 5.0 kJ·m^−2^ treatment maintained higher springiness (10.52 N) than the control (9.78 N), indicating a positive effect on texture retention.

### 3.3. Changes in Membrane Integrity

**Figure 3:**
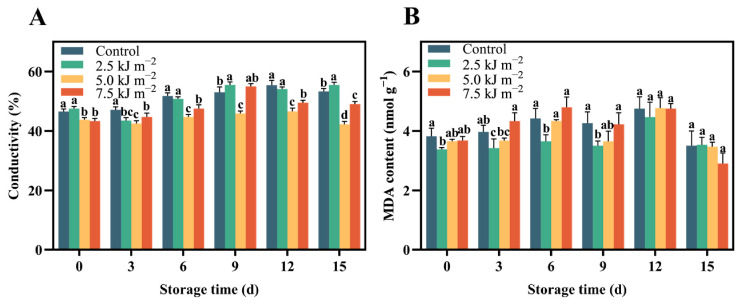
Effects of UV-C treatments on conductivity (**A**) and MDA (**B**) of shiitake mushrooms stored at 0 °C for 15 days. Data are presented as mean ± standard error (*n* = 3). Different lowercase letters above bars indicate significant differences (*p* < 0.05).

The changes in electrical conductivity (EC) and MDA content, two indicators of membrane integrity and lipid peroxidation, are presented in Figure 3A,B, respectively. Regarding EC, all treatment groups showed a gradual increase during the first 12 days of storage, followed by a slight decline at day 15. The control group exhibited EC values ranging from 46.5% (day 0) to 55.4% (day 12), dropping to 53.3% at day 15. Among the UV-C treatments, the 5.0 kJ·m^−2^ group consistently maintained the lowest EC throughout the storage period, with values of 43.8% (day 0), 42.5% (day 3), 44.7% (day 6), 45.9% (day 9), 46.6% (day 12), and 42.2% (day 15). In contrast, the 2.5 and 7.5 kJ·m^−2^ treatments showed higher and more variable EC, reaching 55.5% and 55.0% at day 9, respectively. Similarly, for MDA content, an initial increase was observed in all groups up to day 6 or day 12, followed by a decrease at day 15. The control group peaked at 4.75 nmol/g at day 12, then declined to 3.50 nmol/g at day 15. The 5.0 kJ·m^−2^ treatment showed relatively stable MDA levels throughout storage, ranging from 3.65 to 4.77 nmol/g, while the 7.5 kJ·m^−2^ treatment exhibited the highest MDA at day 6 (4.80 nmol/g) but dropped sharply to 2.90 nmol/g at day 15. Collectively, these results indicate that UV-C irradiation at 5.0 kJ·m^−2^ effectively maintained membrane integrity and mitigated lipid peroxidation in shiitake mushrooms during postharvest storage, as evidenced by lower EC and more stable MDA levels compared to the control and other UV-C treatments.

### 3.4. Changes in Defense Enzyme Activities

**Figure 4:**
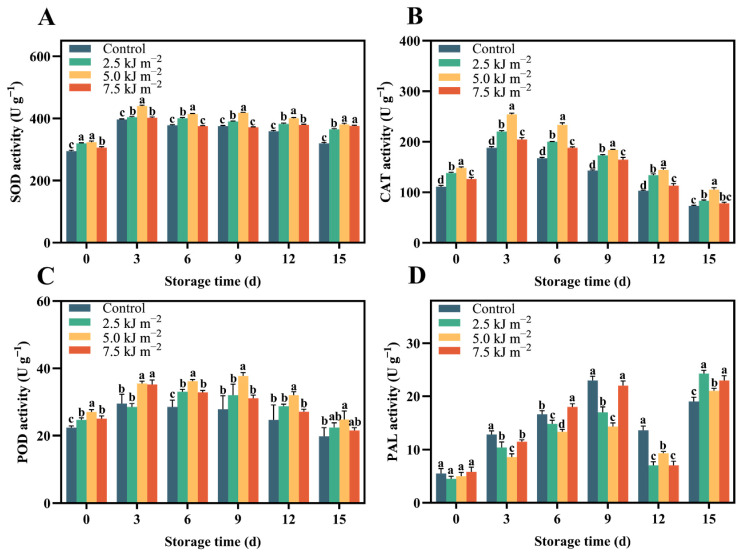
Effects of UV-C treatments on SOD (**A**), CAT (**B**), POD (**C**) and PAL (**D**) of shiitake mushrooms stored at 0 °C for 15 days. Data are presented as mean ± standard error (*n* = 3). Different lowercase letters above bars indicate significant differences (*p* < 0.05).

The activities of defense-related enzymes (SOD, CAT, POD, and PAL) during postharvest storage are presented in Figure 4A–D. Overall, all enzyme activities initially increased and then decreased over time, with the highest values generally observed between day 3 and day 9. For SOD activity, the control group increased from 295 U/g at day 0 to a peak of 397 U/g at day 3, then gradually declined to 320 U/g at day 15. All UV-C treatments maintained higher SOD activities than the control throughout storage, with the 5.0 kJ·m^−2^ treatment showing the most pronounced effect, reaching 440 U/g at day 3 and remaining at 381 U/g at day 15, compared to 320 U/g in the control. Similarly, CAT activity in the control group peaked at 188 U/g at day 3 and dropped to 73 U/g at day 15. The 5.0 kJ·m^−2^ treatment exhibited the highest CAT activity at each time point, with values of 254 U/g (day 3), 233 U/g (day 6), 184 U/g (day 9), 144 U/g (day 12), and 105 U/g (day 15). The 2.5 and 7.5 kJ·m^−2^ treatments showed intermediate levels. Regarding POD activity, the control group peaked at 29.5 U/g at day 3 and decreased to 19.8 U/g at day 15. The 5.0 kJ·m^−2^ treatment consistently displayed the highest POD values, reaching 35.5 U/g at day 3, 36.2 U/g at day 6, 37.8 U/g at day 9, 32.0 U/g at day 12, and 24.8 U/g at day 15. As for PAL activity, which is involved in phenylpropanoid metabolism, all groups showed a gradual increase up to day 9, followed by a decline and then a secondary rise at day 15. The control group increased from 5.5 U/g at day 0 to 23.0 U/g at day 9, then dropped to 13.6 U/g at day 12 before rising again to 19.0 U/g at day 15. Among UV-C treatments, the 7.5 kJ·m^−2^ treatment exhibited the highest PAL activity at day 6 (18.0 U/g) and day 9 (22.0 U/g), while the 5.0 kJ·m^−2^ treatment showed a relatively stable increase, reaching 21.0 U/g at day 15. Collectively, these results demonstrate that UV-C irradiation, particularly at 5.0 kJ·m^−2^, markedly boosted the activities of both antioxidant enzymes and a key defense enzyme (PAL), thereby contributing to improved oxidative stress tolerance and postharvest quality retention in shiitake mushrooms.

### 3.5. Changes in Chitin Metabolism

**Figure 5:**
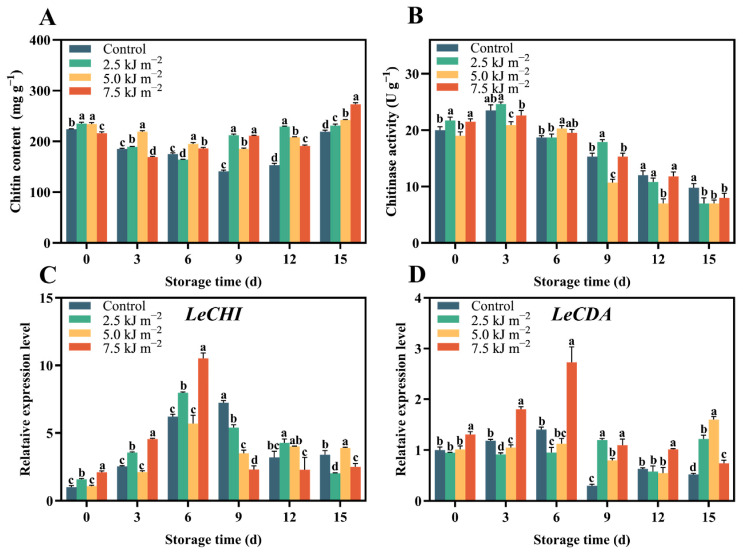
Effects of UV-C treatment on chitin content (**A**), chitinase activity (**B**), *LeCHI* (**C**) and *LeCDA* (**D**) expression level in shiitake mushrooms stored at 0 °C for 15 days. Data are presented as mean ± standard error (*n* = 3). Different lowercase letters above bars indicate significant differences (*p* < 0.05).

Changes in chitin metabolism, including chitin content, chitinase activity, and the relative expression of chitinase (CHI) and chitin deacetylase (CDA) genes, are summarized in Figure 5. Regarding chitin content, the control group decreased from 224 mg/g at day 0 to 141 mg/g at day 9, followed by a relative increase to 219 mg·g^−1^ at day 15, possibly due to cell wall concentration or condensation as other components degraded. Among UV-C treatments, the 5.0 kJ·m^−2^ group maintained relatively stable chitin levels, ranging from 219 mg/g (day 3) to 195 mg/g (day 6) and ending at 242 mg/g at day 15, while the 7.5 kJ·m^−2^ treatment showed the highest final content (273 mg/g at day 15), indicating possible chitin accumulation or reduced degradation. In terms of chitinase activity, a progressive decline was observed during storage. The control group fell from 20.0 U/g at day 0 to 9.8 U/g at day 15, whereas the 5.0 kJ·m^−2^ treatment consistently exhibited the lowest activity after day 3 (20.9, 20.3, 10.7, 7.0, and 7.0 U/g at days 3, 6, 9, 12, and 15, respectively), suggesting suppression of chitin degradation. As for gene expression, CHI transcript levels increased sharply in early storage, peaked at day 6 or 9, and then declined. The control group reached a 6.2-fold peak at day 6, while the 7.5 kJ·m^−2^ treatment showed the highest CHI expression (10.5-fold at day 6). The 5.0 kJ·m^−2^ treatment exhibited a moderate peak (5.7-fold at day 6) but maintained higher expression than the control at day 15 (3.9 vs. 3.4). In contrast, CDA expression was generally low and fluctuated. The control group peaked at 1.4-fold at day 6 and dropped to 0.52 at day 15, whereas the 5.0 kJ·m^−2^ treatment showed a gradual increase towards the end of storage (1.6-fold at day 15), and the 7.5 kJ·m^−2^ treatment exhibited an early peak at day 6 (2.7-fold) followed by a decline. Collectively, these results indicate that UV-C irradiation, particularly at 5.0 kJ·m^−2^, modulates chitin metabolism by preserving chitin content during the early and middle storage stages (days 0–9), suppressing chitinase activity, and upregulating CDA expression at late storage stages, thereby contributing to cell wall integrity and partially delayed softening.

## 4. Discussion

Shiitake mushrooms, which are widely cultivated especially in Asian countries and have seen rapid growth in production and consumption because of their unique flavor and nutritional composition (including polysaccharides, ergosterol, L-ergothioneine, etc.), are nevertheless highly perishable and undergo rapid postharvest quality deterioration. In this study, we found that a UV-C dose of 5.0 kJ m^−2^ effectively delayed quality deterioration of shiitake mushrooms, an effect that may arise from UV-C’s synergistic regulation of respiratory metabolism, cellular structural integrity, and redox homeostasis.

### 4.1. General Mechanisms of UV-C in Postharvest Horticultural Products

Postharvest shiitake mushrooms retain high physiological activity, and vigorous respiration rapidly consumes reserves, leading to weight loss and accelerated metabolic senescence. In our study, 5.0 kJ m^−2^ UV-C irradiation significantly suppressed the respiration rate. Given that respiration is central to energy metabolism, its suppression directly reduces substrate catabolism, thereby slowing dry matter loss [8]. This effect may be attributable to a UV-C–induced mild stress response, which downregulates overall metabolic rate by modulating mitochondrial energy metabolism pathways without inducing severe membrane damage. This result is consistent with reports of suppressed respiration in irradiated grapes [33]. In contrast, higher UV-C doses may disrupt membrane integrity and trigger stress-enhanced respiration, thereby accelerating quality deterioration. This dose-dependent response is consistent with previous findings in *Pholiota adiposa*, a close relative of shiitake mushrooms [34], further highlighting that precise dose optimization is essential to achieve beneficial hormetic effects.

In addition, despite the high moisture content and vigorous postharvest metabolism of shiitake mushrooms, UV-C irradiation at 5 kJ·m^−2^ effectively reduced respiration rate and water loss. The high water activity may slightly scatter UV-C, but the treatment still successfully activated surface-localized defense mechanisms and preserved overall quality, likely because the hormetic response is triggered in the outer cell layers where UV-C is most effective [35]. Regarding color, it is worth noting that although UV-C treatment at 5 kJ·m^−2^ significantly preserved lightness (L*), it did not cause significant changes in a* (redness) or b* (yellowness) values, suggesting that the anti-browning effect of UV-C is primarily manifest as maintenance of surface brightness rather than alteration of hue.

### 4.2. Mushroom-Specific Effects on Cell Wall Integrity

The cell wall is the structural basis for tissue firmness [3,21]. Our results indicate that UV-C treatment not only preserved shiitake firmness but also maintained higher chitin content and inhibited chitinase activity. This suggests that the treatment preserves cell wall integrity by suppressing cell-wall-degrading enzyme activity and slowing hydrolysis of wall polysaccharides. A similar mechanism has been reported in other horticultural products—for example, UV-C delays softening in strawberry by inhibiting cell-wall-degrading enzymes [36,37]. Moreover, our gene expression analysis showed that UV-C altered the expression patterns of key genes involved in cell wall degradation (*LeCHI* and *LeCDA*), thereby supporting cell wall stability at the transcriptional level.

Cell membranes are fundamental to normal fruit and vegetable metabolism, and accumulation of the lipid peroxidation product malondialdehyde (MDA) increases membrane permeability, serving as an indicator of membrane structural damage [21]. In this study, UV-C treatment significantly reduced MDA content and membrane relative electrical conductivity, indicating effective suppression of lipid peroxidation and preservation of membrane integrity. Stabilization of membrane structure not only limited electrolyte leakage but also provided the basis for maintaining redox homeostasis [15]. When reactive oxygen species (ROS) levels exceed the clearance capacity of endogenous antioxidant systems, oxidative stress is triggered; sustained oxidative damage then activates enzyme systems and gene expression associated with browning and softening [14,15]. We demonstrate that UV-C concurrently decreased membrane injury and enhanced activities of antioxidant enzymes, including SOD and CAT, thereby improving the oxidative stress tolerance of *L. edodes*.

It should be noted that, during the later storage period (days 12–15), chitin content showed a relative increase (Figure 5A) despite the continuous decline of hardness. This seemingly contradictory phenomenon can be explained as follows. First, the cell wall of edible fungi is structurally complex, composed not only of chitin but also of chitosan (a deacetylated chitin derivative), β-1,3-glucan, β-1,6-glucan, and mannoproteins [6]. The overall mechanical strength (hardness) depends on synergistic interactions among these components. Even if chitin content recovers or becomes relatively concentrated, the degradation of glucans and the disruption of hyphal entanglement can still lead to a net loss of firmness. Second, the late increase in chitin content (on a dry weight basis) may be a relative concentration effect caused by the loss of other soluble components (sugars, organic acids) and water, rather than true net synthesis of chitin. Third, although chitinase activity declines during storage (Figure 5B), the cumulative damage to the cell wall matrix—especially the glucan network—is largely irreversible. Therefore, the preservation of chitin alone is not sufficient to maintain firmness at very late stages, but early suppression of chitinase activity by UV-C (5 kJ·m^−2^) still contributes to delaying softening during the first 9–12 days.

### 4.3. Dose-Dependent Effects and Oxidative Stress

A higher UV-C dose (7.5 kJ·m^−2^) induces membrane damage via excessive ROS. Although we did not directly measure specific ROS, it is well established that high-dose UV-C can lead to the generation of highly reactive radicals such as hydroxyl radicals (·OH) and superoxide anions (O_2_^−^) [10,14]. These radicals attack polyunsaturated fatty acids in the plasma membrane, initiating chain lipid peroxidation, which explains the significantly higher MDA content and electrolyte leakage observed in the 7.5 kJ·m^−2^ treated samples (Figure 3). Moreover, excessive UV-C can cause physical disruption of surface cells, leading to accelerated water loss (higher weight loss, Figure 2B) despite a transient suppression of respiration. Consequently, the 7.5 kJ·m^−2^ dose exceeds the hormetic zone for shiitake mushrooms, shifting from a protective to a damaging effect. This finding underscores the importance of precise dose optimization.

Crucially, UV-C exerted differential regulation on quality-related enzymes: it inhibited polyphenol oxidase and laccase activities, blocking the conversion of phenolic compounds into melanin and directly suppressing enzymatic browning [38,39]. This dual regulatory pattern—suppressing deleterious enzymes while activating defensive enzymes—reflects UV-C as a physical stressor that precisely elicits protective stress responses rather than causing metabolic disorder through excessive damage. It is important to note the species specificity of such responses; for example, UV-C increases PAL activity in grapefruit [40], underscoring the significance of stress intensity and system compatibility.

In summary, a 5.0 kJ m^−2^ UV-C treatment delayed postharvest senescence of shiitake mushrooms via three interrelated pathways: suppression of respiratory metabolism, maintenance of cellular structural integrity, and reconfiguration of redox balance and enzyme systems. Our study systematically reveals the multi-target preservation mechanisms of UV-C at the physiological and biochemical levels and clarifies the key role of cell wall metabolism in delaying softening of edible fungi, providing a theoretical basis for developing efficient and safe physical preservation technologies. Future research should further elucidate upstream transcriptional regulatory networks of UV-C signaling and validate the treatment’s applicability across diverse edible mushroom cultivars to promote integration of this technology into cold-chain logistics.

## 5. Conclusions

This study systematically examined the effects of varying UV-C irradiation doses on the postharvest quality of shiitake mushrooms. Among the tested doses, 5.0 kJ m^−2^ UV-C significantly delayed weight loss (reduced by ~32% at day 15), suppressed cap opening (27.3% vs. 48.0% in control at day 15) and respiration, and maintained firmness (31.0 N vs. 26.2 N in control at day 15) and surface color (L* preserved, while a* and b* showed no significant changes) of shiitake mushroom. Further mechanistic analysis revealed that 5.0 kJ m^−2^ UV-C irradiation stabilized chitin content and suppressed chitinase activity to maintain cell wall integrity, reduced electrical conductivity and MDA content to alleviate membrane permeability increase and preserve cell membrane integrity, and enhanced the activities of antioxidant enzymes (SOD, CAT, POD, PAL increased 1.5- to 2.8-fold), thus strengthening the mushroom’s antioxidant defense system. This study not only clarifies the preservative effect of UV-C irradiation on shiitake mushrooms but also elucidates its underlying physiological and biochemical mechanisms, providing a scientific basis for developing efficient and ecofriendly postharvest ultraviolet preservation technology. Nevertheless, this study has several limitations: only one cultivar was tested, and flavor compounds or microbial reduction were not analyzed. Future multi-omics studies and pilot-scale trials under commercial conditions are needed to further validate the 5 kJ·m^−2^ dose. Overall, these findings hold significant theoretical reference value and practical implications for extending the storage time of edible fungi and minimizing postharvest losses.

## Data Availability

The original contributions presented in this study are included in the article. Further inquiries can be directed to the corresponding author.

## Abbreviations

The following abbreviations are used in this manuscript:

UV-C	Low-dose ultraviolet-C
MDA	Malondialdehyde
SOD	Superoxide dismutase
CAT	Catalase
POD	Peroxidase
PAL	Phenylalanine ammonia-lyase
